# Identification, Isolation, and In Vitro Culture Trials of Ovarian Germ Stem Cells from Different Teleost Fish Species

**DOI:** 10.3390/vetsci12121179

**Published:** 2025-12-10

**Authors:** Caterina Varvara, Gianluca Ventriglia, Chrysovalentinos Pousis, Teresa Di Gioia, Rosa Zupa, Deborah Maria Del Frassino, Aldo Corriero, Tiziana Martinello

**Affiliations:** 1 Department of Veterinary Medicine, University of Bari Aldo Moro, 70010 Valenzano, Italy; caterina.varvara96@gmail.com (C.V.); deborah.delfrassino@uniba.it (D.M.D.F.); aldo.corriero@uniba.it (A.C.); tiziana.martinello@uniba.it (T.M.); 2 SIAV B Macroarea Nord, Asl Bari, 70037 Ruvo di Puglia, Italy; teresa.digioia@asl.bari.it

**Keywords:** stem germ cells, stemness markers, female germline

## Abstract

Germ cell xenotransplantation is a promising tool for enhancing aquaculture production and supporting the conservation of declining fish species. A major limitation is the reliable identification of germ stem cells, which remains challenging due to the scarcity of validated molecular markers. This study assessed the suitability of different immunohistochemical approaches for identifying ovarian germ stem cells (OGSCs) in three Mediterranean species of commercial interest: European hake (*Merluccius merluccius*), meagre (*Argyrosomus regius*), and flathead grey mullet (*Mugil cephalus*). Among the antibodies tested (anti-OCT4, anti-VASA, anti-Sox2), only anti-VASA and anti-Sox2 labeled cells with morphological features consistent with OGSCs, although staining intensity, specificity, and cross-reactivity with early oocytes varied across species. In parallel, preliminary protocols for OGSC isolation and in vitro proliferation were developed for European hake and meagre. Enzymatic dissociation with collagenase, followed by Ficoll-Paque density separation and culture in Leibovitz’s L-15 medium supplemented with 2% fetal bovine serum at 25 °C, resulted in the highest OGSC recovery and detectable increases in proliferating cells. A sequential pre-plating step enhanced enrichment in meagre but caused substantial cell loss in hake. Overall, this study provides a comparative evaluation of female germline stem cell markers in these species, contributing to future surrogate reproduction and germplasm conservation strategies.

## Simple Summary

Germ cell xenotransplantation, i.e., the production of fertile donor gametes in a host species for which the farming technology is well established, is a promising biotechnology that could significantly contribute to species diversification in aquaculture. However, this method requires critical preliminary steps, such as isolation, characterisation and in vitro proliferation of donor gametes, which depend on several variables and are generally species-specific. In this study, we tested different immunostaining protocols to characterise ovarian germ stem cells in three commercially important fish species, European hake, meagre, and flathead grey mullet, using commonly used stemness markers. We also attempted to establish a protocol for the isolation of ovarian germ stem cells from meagre and European hake and to establish the appropriate conditions for in vitro proliferation. The results show that the efficacy of antibodies against stemness markers may vary according to the fish species, fixative, and antigen retrieval procedure and that the isolation and in vitro proliferation protocols for ovarian germ stem cells should be modulated according to the fish species.

## 1. Introduction

Fish, representing almost half of all known vertebrate species, are the most diverse group of vertebrates [1], largely due to their remarkable reproductive adaptability across a wide range of environmental conditions [2]. Over the past five decades, global per capita fish consumption has almost doubled, equalling the consumption of poultry and pork on an edible weight basis [3,4,5]. Synergistic interactions between human-caused climate change and anthropogenic activities [6,7] have been leading to a depletion of wild stocks, with a large number of species being listed as extinct, extinct in the wild, critically endangered, endangered, vulnerable, or near threatened [8]. Aquaculture represents the fastest-growing sector of marine and freshwater food production, crucial to meeting global fish demand [9], now contributing 56% of the world’s fish supply for direct human consumption [5]. The rapid growth of aquaculture, driven by the expansion in the production of established species and the introduction of new species over the past three decades, has contributed to biodiversity conservation by alleviating pressure on wild stocks [10].

Reproductive techniques, such as germ cell xenotransplantation, i.e., the technology that enables the production of donor-derived gametes through surrogate gametogenesis, have been instrumental in overcoming significant reproductive challenges and advancing aquaculture technology [11]. Due to their plasticity, cryopreserved germ stem cells can be transplanted into the embryos, larvae, and gonads of host species to generate eggs or sperm [11,12,13,14]. Genetically manipulated germ stem cells can also be transplanted to produce genetically modified fish with characteristics such as faster growth or disease resistance [11]. Moreover, this technique can be used to produce valuable species that are difficult to breed using surrogate species whose production is standardised and for germline conservation and propagation of valuable and/or endangered fish species [11].

The selection of recipient species is based on specific characteristics: a relatively smaller body size, a shorter generation time, and a well-established protocol for the control of reproduction [15]. Xenotransplantation consists of different steps, such as the purification of donor stem cells, in vitro propagation, cell labelling and transferring into recipient species, and the identification of donor cells [16]. One of the crucial steps of this biotechnology is represented by the correct identification of the germ stem cells (GSCs), which ensures the selection of viable spermatogonia or oogonia capable of colonising, self-renewing, and differentiating in the recipient gonads [17]. In mammals, a broad range of molecular markers for germline stem cells (GSCs) and their progenitors has been identified, including surface proteins, RNA-binding proteins, zinc-finger proteins, cytokines, and cell-cycle regulators [18,19]. In fish, by contrast, the lack of specific molecular markers still represents a major limitation for the identification and isolation of GSCs [20,21]. Moreover, the identification of stemness markers and stem germ cell purification techniques in fish have focused on the male germ line [22,23], while few studies have focused on the characterisation and purification of the female germ line [24]. In different fish species, it has been shown that spermatogonial stem cells differentiate into oogonia and produce functional eggs when they are transplanted in females, demonstrating the sexual plasticity of germ stem cells [17,22,25]. However, heterogametic sex determination systems have been reported in approximately 10% of fish species, and both male (XY) and female (ZW) heterogametic systems have been described [26,27]. In heterogametic fish species, the transplantation of spermatogonia stem cells in females results in the production of eggs with male genotypes [17], which seriously affects the sex proportion of the progeny. In teleost model species, research on the identification and characterization of ovarian germline stem cells (OGSCs) has progressively expanded in recent years [28,29]. In medaka (*Oryzias latipes*), early-stage germ cells have been shown to localize in discrete clusters distributed along interwoven, thread-like cords composed of sox9b-expressing somatic cells—structures referred to as germinal cradles—within which germ cell development takes place [29]. Nonetheless, significant knowledge gaps remain as specific molecular markers for OGSCs are still poorly defined in most non-model species; the microenvironment of the niche and the mechanisms regulating OGSC fate (self-renewal vs. differentiation) are not well understood; and the ability to culture OGSCs in vitro for long-term expansion and subsequent differentiation into functional oocytes is still largely undeveloped [30].

Although ovarian germline stem cells (OGSCs) hold considerable potential for surrogate reproduction, germplasm preservation, and advanced breeding programs in aquaculture, progress has been constrained by the lack of validated tools for their detection and recovery in non-model species. Most available immunohistochemical markers and isolation protocols have been developed for gonadal stem cells of laboratory fishes or for spermatogonial lineages [22,23], with limited evidence supporting their applicability to female germlines in commercially relevant taxa. The present study addresses this gap by providing the first comparative evaluation of stemness-related antibodies for the identification of self-renewing OGSCs in three Mediterranean fish species of high economic importance: (1) the European hake (*Merluccius merluccius*), an intensely exploited demersal gadiform distributed in the eastern Atlantic and Mediterranean Sea; (2) the meagre (*Argyrosomus regius*), a benthopelagic, oceanodromous species with strong aquaculture potential but restricted production due to limited broodstock availability; and (3) the flathead grey mullet (*Mugil cephalus*), a cosmopolitan mugilid that exhibits severe reproductive dysfunctions in captivity. In parallel, we developed preliminary yet species-specific protocols for OGSC purification and in vitro proliferation in hake and meagre, laying essential groundwork for future applications in surrogate reproduction and germplasm conservation.

## 2. Material and Methods

### 2.1. Fish Sampling

For the present study, male European hake (*N* = 4, mean body mass = 150 ± 1.41 g), flathead grey mullet (*N* = 4, mean body mass = 350 ± 2.45 g), and meagre (*N* = 4, mean body mass = 3500 ± 2.94 g) were used. Wild-caught European hake and flathead grey mullet were purchased upon landing from the wholesale market in Bisceglie (Apulia, Italy) in mid-March 2024. These fish were caught by fishing vessels operating in the South Adriatic Sea, where sea surface temperature and salinity at the time of capture were approximately 13–15 °C and 38.5 PSU, respectively. Hatchery-produced meagre reared in a sea cage and harvested according to routine commercial procedures were purchased from Rheomare InMare S.r.l. (Gallipoli, Apulia, Italy) in mid-April 2024, when sea surface temperature and salinity were approximately 15 °C and 39 PSU, respectively.

The sampled fish were stored in a cold box and transported to the laboratory, where they arrived within one to two hours.

### 2.2. Ovary Histological Analysis and Identification of Ovarian Germ Stem Cells

Ovary cross sections (0.5 cm thick) were taken from each fish and fixed in Davidson’s and Bouin’s solutions, dehydrated in ethanol, clarified in xylene, and embedded in paraffin wax. Four-µm thick sections stained with haematoxylin–eosin (H-E) were used to identify germ cell stages according to the description of Corriero et al. [31].

For the immunohistochemical identification of OGSCs, the following commercial antibodies against stemness antigens were tested: rabbit anti-OCT4 (polyclonal; immunoreactive with the human nuclear antigen Octamer-Binding Protein 4) (Thermo Fisher Scientific, Waltham, MA, USA; PA5-27438), rabbit anti-VASA (polyclonal immunoreactive with the zebrafish cytoplasmic antigen DEAD Box Protein 4) (Abcam, Cambridge, UK; ab209710), and mouse anti-Sox2 (monoclonal; immunoreactive with the human nuclear protein Transcription factor Sox2) (Santa Cruz Biotechnology Inc., Dallas, TX, USA; sc-365823). The amino acid sequences of the peptides used for the immunisation and the similarity of the investigated fish species with the available corresponding amino acid sequences are reported in Supplementary Table S1. For the immunohistochemical analyses, four-µm thick sections were cut, de-paraffinised and rehydrated through graded ethanol solutions. All immunostainings were performed with or without antigen retrieval. For antigen retrieval, ten histological slides were immersed in 100 mL of citrate buffer (0.01 M, pH 6.0) or Tris-EDTA buffer (10 mM Tris, 1 mM EDTA, 0.05% Tween 20, pH 9) and microwaved at full power (750 W) in four 5-min cycles. Fresh buffer was added between cycles in both antigen retrieval procedures. After treatment, the slides were left to cool for 10 min in 100 mL of fresh, new buffer. Endogenous peroxidase activity was inhibited by treating the sections for 10 min with 3% H_2_O_2_ and then rinsing them with distilled water and phosphate-buffer saline (0.01 M, pH 7.4, containing 0.15 M NaCl) (PBS). Subsequently, the sections were incubated for 30 min in normal horse serum (NHS) (Vector Laboratories, Inc., Burlingame, CA, USA) to block nonspecific binding sites for immunoglobulins. The sections were then incubated overnight with primary antibodies diluted in PBS containing 0.1% bovine serum albumin (BSA) (Sigma-Aldrich S.r.l., Milan, Italy) at 4 °C. The following antibody dilutions were tested: anti-VASA, 1:500, 1:1000, 1:2000; anti-OCT4 1:300, 1:500; anti-Sox2 1:300, 1:500. After rinsing for 10 min in PBS, immunohistochemical visualisation was conducted using the Vectastain Universal Elite Kit (Vector Laboratories, Inc., Burlingame, CA, USA). Peroxidase activity was visualised through incubation for 8 min with a Vector DAB (3,30-diaminobenzidine) Peroxidase Substrate Kit (Vector Laboratories, Inc., Burlingame, CA, USA), which produces a brown precipitate. To confirm the specificity of the immunoreaction, several control staining procedures were performed: (i) replacement of the primary antibody with normal horse serum and PBS; (ii) replacement of the primary antibody with an isotype-matched, non-specific antibody (rabbit anti-Caspase 3 IgG; rabbit anti-Histone H3 IgG) to demonstrate that the positive immunoreactions were not due to non-specific reactivity of rabbit IgG; (iii) immunostaining of meagre brain sections with polyclonal rabbit anti-VASA IgG and monoclonal mouse anti-Sox2 IgG to confirm the absence of immunoreactivity in a tissue lacking stem cells; (iv) immunostaining of testis sections from pre-pubertal grey mullet using anti-VASA, anti-OCT4, and anti-Sox2 antibodies to verify the immunoreactivity of the antibodies in a fish tissue known to contain germ stem cells; (v) immunostaining of testis sections from pre-pubertal cats with anti-OCT4 and anti-Sox2 antibodies to assess the cross-reactivity of the two mammalian IgGs in a mammalian tissue containing germ stem cells. The cat testis sections were part of a tissue collection used in a previous study on cat spermatogenesis [32].

### 2.3. European Hake and Meagre Ovarian Germ Stem Cell Isolation and In Vitro Proliferation

Small ovary samples (500 mg) were excised aseptically and washed in PBS solution containing 1% penicillin–streptomycin (Pen/Strep). Tissue samples were then washed and immersed in PBS and minced using small dissecting scissors for cell culture before enzymatic digestion. Subsequently, ovarian fragments were enzymatically dissociated by incubation with either 125 U mL^−1^ type IA collagenase or 0.025% trypsin. In both cases, the digestion lasted for a total of two hours, during which the tissue was gently pipetted every 15 min to facilitate dissociation. The enzyme treatments were performed in an atmosphere containing 5% CO_2_, at either 25 °C or 28 °C. The samples were filtered with a 100 µm cell strainer and centrifuged at 100× *g* for 5 min with a MPW centrifuge (M-diagnostic). The pellet containing germ cells was suspended in 1 mL of PBS for gradient separation. A Ficoll-Plaque PLUS (GE Healthcare Life Sciences, Uppasala, Sweden) gradient was prepared in a 15 mL centrifuge tube. To achieve this, 1 mL of cell suspension was gently added to 5 mL of Ficoll–Paque PLUS solution. After centrifugation at 800× *g* for 30 min at room temperature, with brake and acceleration set to the lowest value (0), the cell suspension separated into two distinct phases with a single interphase band in between. The interphase band, which contained the target cells, was carefully collected for further processing. The cells were then re-suspended either in the culture medium used in our previous study [33] for meagre spermatogonia in vitro proliferation (Medium A) or in Leibovitz’s L-15 (L-15) (Sigma-Aldrich, St. Louis, MO, USA) supplemented with 2% fetal bovine serum (FBS, Sigma-Aldrich) and 1% penicillin–streptomycin (Pen/Strep, Sigma-Aldrich) (Medium B), a medium successfully used in previous studies on fish stem cell culture [33,34]. The cells were plated in a 24-well plate for in vitro proliferation experiments. Each tissue sample was divided into five parts, which were used to evaluate the presence of OGSCs immediately after isolation (T_day0_), after three days of culture (T_day3_), and after sequential pre-plating (PP-T_day1_, PP-T_day2_, PP-T_day3_) (Supplementary Figure S1). The sequential pre-plating method is based on the different adhesion properties: 24 h after the initial plating, non-adherent cells were collected and transferred to a new well. This process was repeated three times at 24 h interval, allowing the separation and subsequent analysis of both adherent and non-adherent cell fraction at each step (Supplementary Figure S1). Cultured cells were observed under phase contrast microscopy (Nikon Eclipse, Ti-U, Amstelveen, The Netherlands; 400× magnification) and incubated at 25 °C and 28 °C in an atmosphere of 5% CO_2_.

### 2.4. European and Hake Meagre Ovarian Germ Stem Cell Characterisation

For the immunocytochemical identification of OGSCs, adherent and non-adherent cells from T_day0_, T_day3,_ and pre-plating were collected and fixed with 4% paraformaldehyde in a 15 mL centrifuge tube (Becton Dickinson Falcon, Franklin Lakes, NJ, USA) for 20 min; after two rinses in PBS, the fixed cells were divided into equal aliquots and placed at the centre of four microscope slides. The immunolabelling of isolated OGSCs was performed with anti-VASA and anti-Sox2 antibodies. Since the anti-OCT4 antibody did not provide useful results in immunohistochemical staining (see Section 3), this antibody was not used to label OGSCs. The staining protocol was similar to the one described for immunohistochemistry, with some changes: (i) the cells were directly fixed with 4% paraformaldehyde; (ii) the antibody dilution was anti-VASA 1:500; anti-Sox2 1:300; (iii) before incubation in NHS, the fixed cells were incubated from 30 min in a permeabilising solution of 0.3% Triton-X-100 in PBS.

Moreover, to evaluate the increase in cells obtained from the different pre-plating conditions and time points, a subset of fixed cells was stained with H-E, and cell density (cells per mm^2^) was estimated in five visual fields. Images of H-E-stained slides were captured using a 20× objective with a digital camera (DFC 420; Leica Microsystems, Cambridge, UK) mounted on a light microscope and analyzed using an image-analysis software (Leica Application Suite X, version 5.1.0.25446, Wetzlar, Germany). The cell density results were expressed as means ± standard deviation, and statistical differences for the following comparisons: T_day0_ vs. T_day3_ (adherent + non-adherent), T_day0_ vs. PP-T_day1_ (adherent + non-adherent), PP-T_day1_ adherent vs. PP-T_day1_ non-adherent, and T_day3_ adherent vs. T_day3_ non-adherent, were assessed using Student’s *t*-test with a significance level of *p* < 0.05, performed via the built-in statistical function in Microsoft Excel.

## 3. Results

### 3.1. Ovary Histology and Immunohistochemical Labelling of Ovarian Germ Stem Cells

**Figure 1:**
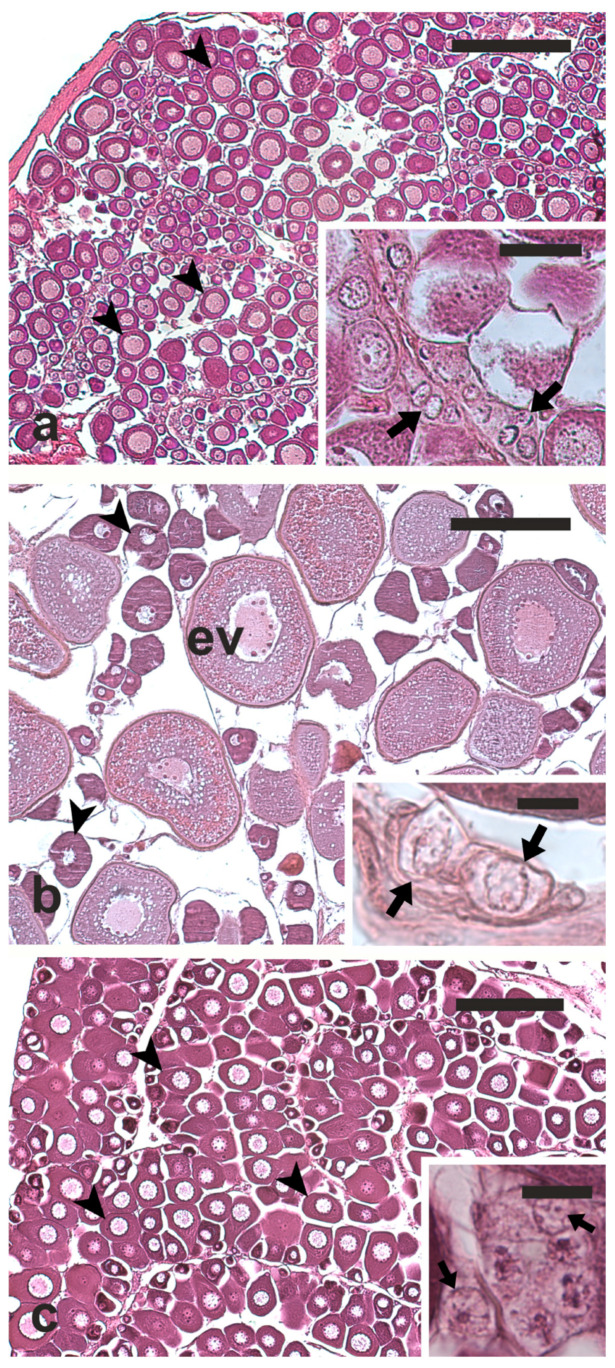
Micrographs of ovary sections from European hake (**a**), meagre, (**b**) and flathead grey mullet (**c**). Arrow, OGSC/oogonium; arrowhead, perinucleolar oocyte; ev, early vitellogenic oocyte. Magnification bars = 200 μm in (**a**–**c**); 30 μm in inset of (**a**), 10 μm in insets of (**b**,**c**).

The histological analysis showed that the ovaries of European hake and flathead grey mullet were inactive, as they had perinucleolar oocytes as the most advanced oocyte stage (maximum oocyte diameter = 60 μm in European hake and 90 μm in flathead grey mullet) (Figure 1a,b). Meagre ovaries were at early gametogenesis stage, since they showed oocytes that had just entered the secondary growth (early vitellogenesis; maximum oocyte diameter = 200 μm) (Figure 1c). In all the examined samples, small clusters of germ cells were observed at the edge of the ovigerous lamellae. These cells, likely OGSCs and oogonia, were round or oval, with a diameter ranging between 8 and 13 µm in the European hake and flathead grey mullet, and between 8 and 15 µm in meagre, and they showed an euchromatic nucleus (Figure 1a–c).

**Figure 2:**
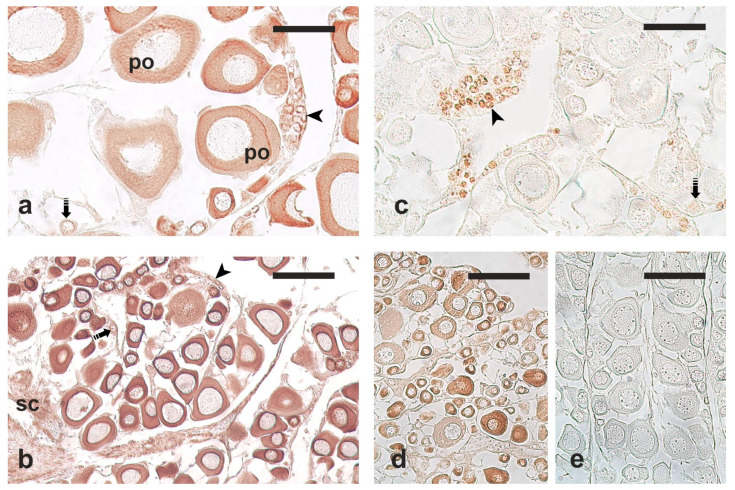
Example micrographs of European hake ovary sections immunostained with antibodies against stem cell markers. (**a**) Anti-VASA; antigen retrieval procedure. (**b**) Anti-VASA; no antigen retrieval procedure. (**c**) Anti-Sox2; no antigen retrieval procedure. (**d**) Anti-OCT4, non-specific ooplasm staining. (**e**) Negative control (primary antibody replaced by phosphate-buffer saline). Arrowhead, ovarian germ stem cells (OGSCs); dashed arrow, chromatin nucleolus oocyte; po, perinucleolar oocyte; sc, somatic (connective) cells. Magnification bars: 50 μm in (**a**,**c**), 100 μm in (**b**,**d**,**e**).

**Figure 3:**
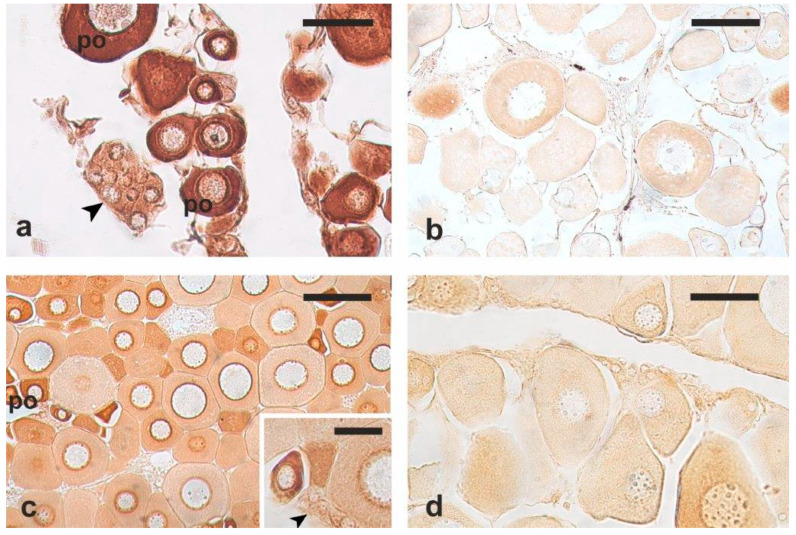
Example micrographs of meagre and flathead grey mullet ovary sections immunostained with antibodies against stem cell markers. (**a**) Meagre; anti-VASA; no antigen retrieval procedure. (**b**) Meagre; anti-Sox2; no antigen retrieval procedure. (**c**) Flathead grey mullet; anti-VASA; antigen retrieval procedure. (**d**) Flathead grey mullet; anti-Sox2; no antigen retrieval procedure. Arrowhead, cluster of ovarian germ stem cells (OGSCs); po, perinucleolar oocyte. Magnification bars: 40 μm in (**a**), 100 μm in (**b**,**c**), 50 μm in (**d**), and 25 μm in inset.

The results of the immunohistochemical labelling are summarised in Table 1. These findings were validated through control immunohistochemical procedures (Supplementary Table S2 and Supplementary Figures S2 and S3). Among the three tested antibodies, only anti-Sox2 and anti-VASA successfully labelled cells that corresponded in size and appearance to the OGSCs/oogonia observed in H-E-stained sections (Figure 2 and Figure 3). Hereafter, these cells are referred to as OGSCs.

The anti-OCT4 antibody did not lead to specific positive immunostaining in any of the investigated species (Figure 2d). The most effective dilution for the anti-Sox2 antibody was 1:500, while the anti-VASA antibody showed the best staining pattern at the dilution of 1:1000. The anti-VASA immunolabelling was more effective when the antigen retrieval procedure was applied, as opposed to the anti-Sox2 antibody whose immunoreactivity was lost after the application of the antigen retrieval procedure.

In European hake, anti-Sox2 antibodies proved to be the most successful in specific labelling of the nuclei of OGSCs, although they also weakly labelled oocytes at the chromatin nucleolus stage. Anti-VASA antibodies marked clusters of OGSCs; however, positivity was also observed in chromatin nucleolar and perinuclear oocytes and, when the antigen retrieval procedure was not applied, also in connective cells.

In meagre, anti-VASA antibodies successfully labelled OGSCs in both samples fixed with Bouin’s solution and Davidson’s solutions; however, positive immunostaining persisted in chromatin nucleolus and perinuclear oocytes. Anti-Sox2 antibodies resulted in no staining in the Bouin’s-fixed samples and non-specific staining in the Davidson’s-fixed samples.

In flathead grey mullet, only anti-VASA antibodies provided specific staining; however, they labelled both OGSCs and primary growth oocytes. Interestingly, the staining intensity decreased with oocyte growth. A weak diffuse non-specific cytoplasmic staining was obtained with anti-Sox2 antibodies.

### 3.2. European Hake and Meagre Ovarian Germ Stem Cell Isolation and In Vitro Culture

In both species, the cell suspension centrifugation in the Ficoll–Paque PLUS density gradient, obtained from tissue samples digested with type IA collagenase at 25 and 28 °C, resulted in a unique band containing several cell types, whereas the use of trypsin resulted in tissue over-degradation and was, therefore, disregarded. In the Ficoll gradient band, a cell type showing morphology and size corresponding to the OGSCs/oogonia identified in the H-E- and IHC-stained sections was identified. Among the two culture media used, Medium B obtained both somatic cells and OGSCs/oogonia, whereas Medium A resulted in an excessive proliferation of somatic cells. The application of the protocol that utilised type IA collagenase as the digestive enzyme and Medium B obtained about 77 and 24 cells mg^−1^ of the sampled tissue in the European hake and meagre samples, respectively.

**Figure 4:**
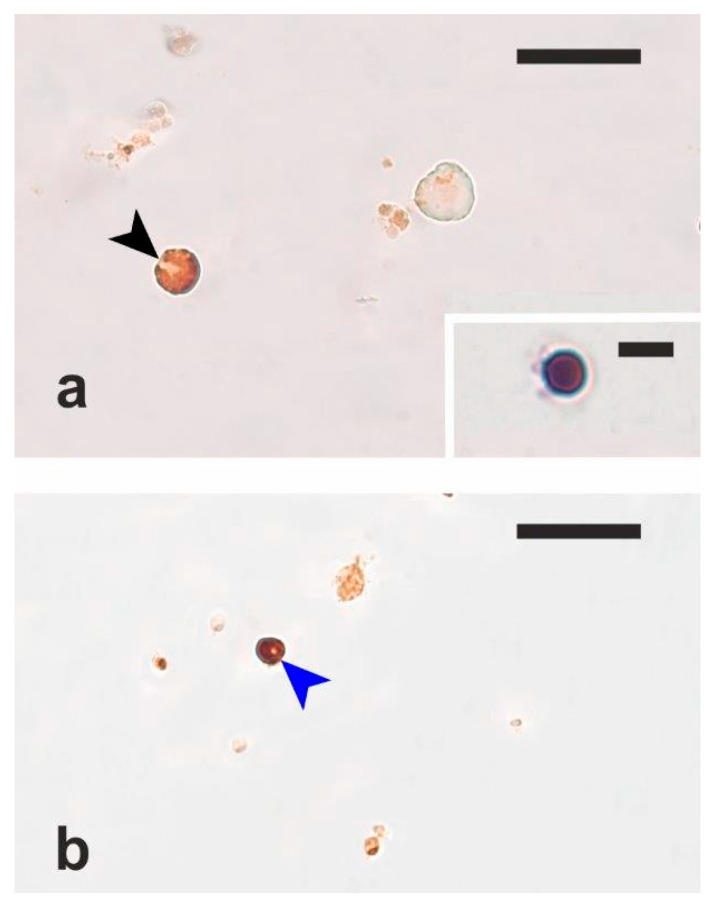
Micrographs of isolated ovarian germ stem cells (OGSCs) (arrowheads). (**a**) Meagre OGSC immunostained with anti-VASA antibodies. (**b**) European hake OGSC immunostained with anti-Sox2 antibodies. Inset of (**a**) shows an isolated OGSC stained with H-E. Magnification bars: 40 μm in (**a**,**b**), 10 μm in inset.

The immunocytochemical analysis showed that the anti-VASA and anti-Sox2 antibodies respectively labelled the cytoplasm and the nucleus of cells with a high nucleus/cytoplasm ratio and diameter ranging between 8 and 13 µm in the European hake and between 8 and 15 µm in the meagre (Figure 4). These cells were subsequently confirmed to be OGSCs.

Cells whose diameter corresponded to that of OGSCs were observed at the time of isolation and after in vitro culture, with and without pre-plating (Supplementary Figure S4). The density (number of cells/mm^2^ microscope slide) of presumptive OGSCs obtained in the different experiments is reported in Table 2. In European hake, OGSC density did not change after pre-plating, and it was significantly reduced after three days without pre-plating. In this species, only a minor fraction of OGSCs were found to be adherent to the well. In meagre, the OGSC density increased significantly both after pre-plating and after three days without pre-plating. In this species, OGSCs did not show a preferential behaviour regarding the tendency to adhere or not to adhere to the well. In both species, sequential pre-plating resulted in the loss of almost all OGSCs; therefore, cell density was negligible in the PP-T_day2_ and PP-T_day3_ samples.

## 4. Discussion

In xenotransplantation experiments, the purity of germ stem cells can affect success rates. Of the many gonadal cell types, only germ stem cells have the capacity to colonise the recipient gonad, proliferate, and differentiate into gametes [11,15]. Therefore, correctly identifying and purifying germ stem cells is a critical step in establishing a surrogate gametogenesis protocol. In the present study, antibodies against markers commonly associated with pluripotent properties of stem cells were used; however, they showed species-specific suitability for labelling OGSCs in immunohistochemical assays. VASA protein, a phylogenetically conserved DEAD-box protein with ATP-dependent RNA-helicase activity [35,36], was first discovered in fruit fly (*Drosophila melanogaster*) through genetic screening, and it was later identified in all vertebrate classes, including fish [37]. In the present study, anti-VASA antibodies were effective but not strictly specific, as they marked both OGSCs and oocytes at early primary growth stage. Incidentally, this also suggests that the immunostaining did not discriminate between OGSGs and oogonia. This has been already reported for the white sturgeon (*Acipenser transmontanus*) [38], and it is not surprising considering that the VASA is required for germ cell maintenance and function [39]. Furthermore, the vasa gene has been reported to be expressed in all stages of gametogenesis [15]. The species-specific immunoreactivity is likely related to the amino acid homology between the peptide sequence used for the antibody production and that of the species being tested. Unfortunately, the VASA amino acid sequences of the three fish species investigated in the present study are not available; however, the identity and positivity between the peptide sequence used to generate the anti-zebrafish VASA antibodies and the corresponding sequence of the meagre congener Japanese meagre (*Argyrosomus japonicus*), were 81% and 91%, respectively (Supplementary Table S1). Interestingly, VASA antibodies were successfully used to label meagre spermatogonia [33].

OCT4 is a transcription factor also known as Oct-3, Oct-3/4, Otf3 or NF-A3, encoded by the Pou5f1 gene and belonging to the POU (Pit, Oct, Unc) family of DNA binding-proteins [40]. This protein, initially identified in mice as a transcription factor specifically expressed in embryonic stem cells and germline cells [41], has been proved to be phylogenetically conserved in vertebrates [42]. Based on sequence homology, the anti OCT4 antibody used in the present study is expected to react with different mammal species (https://www.thermofisher.com/antibody/product/OCT4-Antibody-Polyclonal/PA5-27438 (accessed on 15 May 2025)), but no information is provided by the manufacturer regarding its suitability in fish species. OCT4 amino acid sequences are not available for any of the three analysed fish species. However, it may be hypothesised that the non-specific cytoplasm staining is related to the low similarity among the amino acid sequences used for antibody production and those of the analysed species. Moreover, the non-specific cytoplasm staining might be due to a wide number of amino acids used for immunisation (Supplementary Table S1). Further studies combining immunohistochemical data with molecular analyses may be needed to fully explain the present results.

Sox2, an important transcription and pluripotency factor [43], was reported in mammals like human, mouse, sheep and rabbit, in chicken, in African clawed frog (*Xenopus laevis*), and finally, in teleost fish, such as fugu (*Fugu rubripes*) and zebrafish [43]. Sox2, is a highly expressed protein in proliferating spermatogonial stem cells of rohu carp (*Labeo rohita*), suggesting that it is involved in the regulatory network for the maintenance and proliferation of spermatogonial stem cells [44]. In our study, anti-Sox2 antibodies labelled OGSCs and chromatin-nucleolus stage oocytes, but only in the European hake. Incidentally, as for anti-VASA antibodies, this implicates that anti-Sox2 antibodies did not discriminate between OGSGs and oogonia. The human amino acid sequence used to generate anti-Sox2 antibodies showed a good degree of similarity and positivity (77% in both cases) with flathead grey mullet, the only species among the three investigated for which the amino acid sequence is available (Supplementary Table S1). However, this monoclonal antibody was effective only in the European hake, probably because it is a monoclonal antibody directed against a specific epitope that might not be phylogenetically conserved. Among the fixatives tested in the present study, Davidson’s solution proved to be the most effective, in agreement with previous reports highlighting the limitations of Bouin’s fixative for immunohistochemical applications [45,46]. However, the success of different fixatives may be influenced by the specific target epitope. In particular, formalin-based fixatives are still considered the “gold standard” for histological preservation due to their broad compatibility with various staining and labelling techniques [45,46,47]. Regarding the use of antigen retrieval methods, the potential for unexpected and non-specific immunolabelling must be considered. These methods can unpredictably alter the immunogenic properties of paraffin-embedded tissue, which can be a significant challenge, particularly when working with proteins of unknown localisation, as it becomes difficult to distinguish between specific and non-specific labelling, leading to false-positive results [48]. Therefore, if antigen retrieval is not essential, it is advisable to avoid its use, as demonstrated by the results obtained in European hake, where the anti-Sox2 antibody did not produce any immunostaining in samples fixed with Bouin’s or Davidson’s solution when antigen retrieval was applied, whereas immunoreactivity was observed in the absence of antigen retrieval. A similar pattern was observed in flathead grey mullet, where no immunostaining was detected following the antigen retrieval procedure.

The present study included attempts to purify OGSCs from ovaries containing other germ cells stages and set up a protocol for in vitro proliferation of European hake and meagre OGSCs. Although spermatogonial germ stem cells in several fish species have been shown to be able to differentiate into oogonia when they are transplanted in females [14,22,25], protocols to isolate and culture OGSCs can be particularly useful in fish with heterogametic sex determination systems to avoid a disproportionate sex ratio in the progeny. A series of experiments were carried out under different conditions, including temperature, digestion enzymes, FBS concentrations, and the presence or absence of growth factors to establish the best culture conditions. Trypsin and collagenase are the most commonly used enzymes for gonad dissociation in fish [24] and, in general, the selection of the most appropriate enzyme depends on several variables such as species, sex, enzyme concentration, and exposure time [24]. For ovaries, trypsin was found to be the best enzyme for the digestion of Dabry’s sturgeon (*Acipenser dabryanus*) ovarian tissue [49], while enzymatic dissociation was successfully performed using collagenase in rainbow trout (*Oncorhynchus mykiss*) and zebrafish [17,24,50]. Type IA collagenase at 28 °C has been successfully used to dissociate testes and isolate spermatogonial stem cells (SSCs) in meagre [33]. In the present study, the best results were obtained for both meagre and European hake with type IA collagenase digestion at the temperature of 25 °C.

The isolation and in vitro proliferation of OGSCs from fish ovaries is considered to be more challenging than that of fish SSCs [24]. In the present study, OGSCs/oogonia from the three investigated species were first identified on H-E-stained histological sections based on their morphological features and then their mean diameter was estimated. This procedure allowed us to better identify OGSCs in the subsequent steps of the experiment. The purification of OGSCs in meagre and European hake ovaries was carried out using a Ficoll density gradient, which is commonly used for the isolation and purification of fish SSCs [24]. The nature of the purified cells as OGSCs was confirmed through immunocytochemical staining with anti-VASA and anti-Sox2 antibodies.

It is well known that the Ficoll density gradient does not enable the separation of germ cells from somatic cells, which can overgrow in in vitro proliferation assays and limit the proliferation of germline stem cells; therefore, further steps after gradient density centrifugation may be required to improve the purification of germline stem cells [24]. In the present study, an attempt to further purify OGSCs from meagre and European hake ovaries was made through the pre-plating of previously isolated cells. In general, the results show that a single pre-plating step was sufficient to improve the purification and isolation of OGSCs, whereas 2–3 sequential pre-plating steps reduced the density of OGSCs in culture. In meagre, the density of OGSCs increased over time in both the pre-plating and non-pre-plating procedures. In European hake, on the contrary, a significant increase in OGSC density was observed when the pre-plating procedure was applied. Among post-centrifugation steps, differential plating is considered an effective method to achieve high germ cell purity without causing damage to the isolated cells [15,24]. In medaka (*Oryzias latipes*), Matrigel was the most effective compound for the enrichment of OGSCs by differential plating compared to gelatin, laminin, fibronectin, and poly-l-lysine [15]. Therefore, given the variability of the results obtained for the two species in the present study, the use of Matrigel or other molecules in differential plating could be considered in future experiments on both species. In addition, although differential plating is an effective method of achieving high purity in germ cells, enrichment typically takes 5–7 days, and cell properties may change during the in vitro culture period [24].

The culture medium used for in vitro proliferation of OGSCs in the present study included FBS. An association between a high concentration of FBS and the mitotic activity of somatic cells has been reported for fish germ cell culture [24,49]. Additionally, a significant increase in germ cells mitotic activity was observed in Dabry’s sturgeon with 5% FBS [49]. Moreover, replacing FBS with other factors and/or adding growth factors, such as glial cell line-derived neurotrophic factor (GDNF) and leukaemia inhibitory factor (LIF), as well as adding fish serum to the culture media, could remarkably increase germ cell mitotic activity and decrease somatic cell proliferation [49]. Indeed, the protocol reported by [33] for meagre male germline highlights the importance of using growth factors, such as GDNF, to stimulate SSC proliferation. In the present study, 2% FBS without growth factors (Medium B), and 10% FBS together with several growth factors (Medium A) were tested. Although Medium B was the best in terms of reducing somatic cell proliferation, other suitable growth factors that could promote the proliferation of female germ line stem cells should be investigated. Incidentally, the use of L-15 medium in a CO_2_-enriched atmosphere in fish stem cell cultures is justified by the lower incubation temperature (25 °C) compared with mammals, which reduces acidification caused by CO_2_, and the slower metabolism which results in lower production of acidic metabolites.

Further research is needed to improve OGSC identification and culture, the use of differential plating substrates to enhance OGSC enrichment while minimizing somatic contamination, the testing of species-specific combinations of growth factors and extracellular matrix components, and to confirm the stem nature of the ovarian germ cells identified in the present study.

## 5. Conclusions

This study provides the first comparative evaluation of stemness markers for OGSCs in three fish species of commercial interest, highlighting species-specific differences in antibody performance. Preliminary protocols for collagenase-based isolation and in vitro proliferation in European hake and meagre demonstrate the feasibility of recovering female germline stem cells, laying the groundwork for future applications in surrogate reproduction and germplasm conservation.

## Figures and Tables

**Table 1:** Immunostaining patterns of VASA (cytoplasmic antigen), OCT4 (nuclear antigen), and Sox2 (nuclear antigen) antibodies in the ovaries of the investigated species.

European Hake				
Antibody	Fixative	Antigen Retrieval	No Antigen Retrieval	OGSCs Diameter (Mean ± sd)
VASA Tested dilutions: 1:500; 1:1000; 1:2000	Bouin’s solution	OGSCs; chromatin nucleolus and perinucleolar stage oocytes (best dilution 1:1000).	OGSCs; chromatin nucleolus and perinuclear oocytes; somatic (connective) cells (best dilution 1:1000).	10.3 ± 1.6
	Davidson’s solution	OGSCs; chromatin nucleolus and perinucleolar stage oocytes (best dilution 1:1000).	OGSCs; chromatin nucleolus and perinuclear oocyte; somatic (connective) cells (best dilution 1:1000).	
OCT4 Tested dilutions: 1:300; 1:500	Bouin’s solution	No staining	No staining	
	Davidson’s solution	No staining	No staining	
Sox2 Tested dilutions: 1:300; 1:500	Bouin’s solution	No staining	OGSCs; chromatin nucleolus stage oocytes (best dilution 1:500).	
	Davidson’s solution	No staining	OGSCs; chromatin nucleolus stage oocytes (best dilution 1:500).	
Meagre				
Antibody	Fixative	Antigen retrieval	No antigen retrieval	OGSCs diameter (mean ± sd)
VASA Tested dilutions: 1:500; 1:1000; 1:2000	Bouin’s solution	OGSCs; chromatin nucleolus and perinucleolar stage oocytes (best dilution 1:1000).	OGSCs; chromatin nucleolus and perinucleolar stage oocytes (best dilution 1:1000).	12.4 ± 1.7
	Davidson’s solution	OGSCs; chromatin nucleolus and perinucleolar stage oocytes (best dilution 1:1000).	OGSCs; chromatin nucleolus and perinucleolar stage oocytes (best dilution 1:1000).	
OCT4 Tested dilutions: 1:300; 1:500	Bouin’s solution	Non-specific cytoplasm staining	Non-specific cytoplasm staining	
	Davidson’s solution	Non-specific cytoplasm staining	No staining	
Sox2 Tested dilutions: 1:300; 1:500	Bouin’s solution	No staining	No staining	
	Davidson’s solution	Non-specific cytoplasm staining	Non-specific cytoplasm staining	
Flathead grey mullet				
Antibody	Fixative	Antigen retrieval	No antigen retrieval	OGSCs diameter (mean ± sd)
VASA Tested dilutions: 1:500; 1:1000; 1:2000	Bouin’s solution	OGSCs; chromatin nucleolus and perinucleolar stage oocytes * (best dilution 1:1000).	OGSCs; chromatin nucleolus and perinuclear oocytes; somatic (connective) cells (best dilution 1:1000).	9.9 ± 1.2
	Davidson’s solution	OGSCs; oocytes at chromatin nucleolus and perinucleolar stages * (best dilution 1:1000).	OGSCs; chromatin nucleolus and perinuclear oocytes, somatic (connective) cells (best dilution 1:1000).	
OCT4 Tested dilutions: 1:300; 1:500	Bouin’s solution	No staining	No staining	
	Davidson’s solution	No staining	No staining	
Sox2 Tested dilutions: 1:300; 1:500	Bouin’s solution	No staining	Weak diffuse cytoplasmic non-specific staining	
	Davidson’s solution	No staining	Weak diffuse cytoplasmic non-specific staining	

* The intensity of the immunostaining decreases with oocyte growth.

**Table 2:** Comparison of OGSC density on microscopy slides after isolation and after in vitro culture.

	OGSC Density (Mean ± sd) ^§^	
	European Hake	Meagre
T_day0_ vs. PP-T_day1_ (total)	10.41 ± 6.35 vs. 8.42 ± 2.39 (*p* = 0.47)	3.26 ± 1.17 vs. 13.04 ± 3.41 (*p* < 0.05)
T_day0_ vs. T_day3_ (total)	10.41 ± 6.35 vs. 5.52 ± 4.14 (*p* < 0.05)	3.26 ± 1.17 vs. 8.24 ± 1.21 (*p* < 0.05)
PP-T_day1 (non-adherent)_ vs. PP-T_day1 (adherent)_	7.79 ± 2.69 vs. 0.63 ± 0.40 (*p* < 0.05)	6.88 ± 1.82 vs. 6.16 ± 0.49 (*p* = 0.59)
T_day3 (non-adherent)_ vs. T_day3 (adherent)_	3.98 ± 3.22 vs. 1.54 ± 1.38 (*p* = 0.12)	2.44 ± 1.30 vs. 5.79 ± 1.90 (*p* = 0.07)

^§^ OGSC density is shown as mean ± sd of four biological replicates (five slides per replicate). Student’s *t*-test (statistical acceptance at *p* < 0.05).

## Data Availability

The original contributions presented in this study are included in the article/Supplementary Materials. Further inquiries can be directed to the corresponding author.

## Supplementary Materials

The following supporting information can be downloaded at: https://www.mdpi.com/article/10.3390/vetsci12121179/s1. Supporting information are available as Supplementary Tables S1 and S2 and Supplementary Figures S1–S4. **Table S1**. Comparison between the sequence used for antibody production and the corresponding sequence of the target species available on NCBI. **Table S2**. Immunostaining control procedures. **Figure S1**. Workflow illustrating the procedure for isolation and culture of ovarian germ stem cells (OGSCs), and sequential pre-plating. Ovarian tissue samples (500 mg) were enzymatically dissociated, filtered, and subjected to density gradient separation using Ficoll–Paque PLUS. The recovered cells were plated in 24-well plates and assessed at different time points: immediately after isolation (Tday0) and after three days of culture (Tday3). To enrich OGSCs, sequential pre-plating was performed based on differential adhesion characteristics: non-adherent cells were transferred into new wells at 24-h intervals for three consecutive days (PP-Tday1, PP-Tday2, PP-Tday3). At each stage, adherent and non-adherent cell fractions were separately collected for subsequent analyses. **Figure S2**. Control stainings. a) Rabbit IgG anti-caspase 3 (dilution 1:1000). Negative immunostaining of grey mullet ovary. b) Rabbit IgG anti-caspase 3 (dilution 1:1000). Negative immunostaining of European hake ovary. c) Rabbit IgG anti-Histone H3 (dilution 1:1000). Negative immunostaining of grey mullet ovary. d) Meagre brain (ventral lobes of hypothalamus) stained with haematoxylin-eosin (for comparison with Figure S2e). e) Mouse IgG anti-Sox2 (dilution 1:500). Negative immunostaining of meagre brain. Arrowheads: neuronal perikarya in the periventricular lobe of the hypothalamus. III, cavity of the third ventricle. Magnification bars= 150 µm in a), b), and c); 500 µm in d) and e). **Figure S3**. Control stainings. a) Rabbit IgG anti-VASA (dilution 1:1000). Positive immunostaining of spermatogonial stem cells in seminiferous tubules of juvenile grey mullet. b) Rabbit IgG anti-OCT4 (dilution 1:100). Negative immunostaining of spermtogonial stem cells in seminiferous tubules of juvenile grey mullet. c) Mouse IgG anti-Sox2 (dilution 1:50). Negative immunostaining of spermatogonial stem cells in seminiferous tubules of juvenile grey mullet. d-e) Rabbit IgG anti-OCT4 (dilution 1:250). Positive immunostaining of spermatogonial stem cells in the testis of a pre-pubertal cat. f) Mouse IgG anti-Sox2 (dilution 1:100). Positive immunostaining of spermatogonial stem cells and some peritubular cells in the testis of a pre-pubertal cat. Magnification bars = 150 μm in a)-c); 200 μm in d); 100 μm in e)-f). Arrowheads: immunopositive nuclei of stem spermatogonia. Arrow: immunopositve nucleus of a peritubular cell. **Figure S4**. Phase-contrast microscopy micrograph showing cell populations during pre-plating and culture. a) Freshly isolated cells (T0); b) pre-plating before the isolation of non-adherent and adherent cells (PP-Tday1); c) second pre-plating before isolation of non-adherent and adherent cells (PP-Tday2); d) third pre-plating before isolation of non-adherent and adherent cells (PP-Tday3); e) cultured cells maintained for 3 days post-isolation (T3). Magnification bars: 30 µm. Arrows: putative ovarian germ stem cells (OGSCs).
