# Supplementary material for: Identification, Isolation, and In Vitro Culture Trials of Ovarian Germ Stem Cells from Different Teleost Fish Species
Source: Vet Sci. 2025 Dec 10;12(12):1179. doi: 10.3390/vetsci12121179 (PMC12737627; doi:10.3390/vetsci12121179)
Supplement: Supplementary file 1 [file vetsci-12-01179-s001.zip › Supplementary files/Supplementary Table S1.pdf]

Supplementary Table S1. Comparison between the sequence used for antibody production and the corresponding sequence of the target species available on NCBI.

Rabbit anti-VASA/VAS polyclonal antibody (Abcam, ab209710): recombinant fragment within zebrafish VASA/VAS aa 199-526 (in grey).

1) *Merluccius merluccius*: VASA sequence not available

2) *Argyrosomus japonicus** vs. *Danio rerio**:* 76% identity and 86% positivity

```
Argy.           ----------------------------------------------SDGDRPRVTYIPPT
Danio           GGFRGGFRDGGGDESGKRGFGRGGFRGRNEEVFSKVTTADKLDQEGSENAGPKVVYVPPP
                                                              *:.  *:*.*:**.

Argy.           LPEDEESIFAHYKTGINFDKYDDIMVDVSGTNPPQAIMTFDEAALCESLRKAVTKSGYVK
Danio           PPEEESSIFSHYATGINFDKYDDILVDVSGSNPPKAIMTFEEAGLCDSLSKNVSKSGYVK
                 **:*.***:** ***********:*****:***:*****:**.**:** * *:******

Argy.           PTPVQKHGIPIISAGRDLMACAQTGSGKTAAFLLPILQQLMTDGAAASSFSELQEPEVII
Danio           PTPVQKHGIPIISAGRDLMACAQTGSGKTAAFLLPILQRFMTDGVAASKFSEMQEPEAII
                **************************************::****.***.***:****.**

Argy.           VAPTRELINQIYMEARKFSYGTCVRPVVVYGGVSTGHQIREISRGCNVLCGTPGRLLDVI
Danio           VAPTRELINQIYLEARKFAYGTCVRPVVVYGGINTGYTIREVLKGCNVLCATPGRLHDLI
                ************:*****:*************:.**: ***: :******.***** *:*

Argy.           GRGKIGLSKLRYFVLDEADRMLDMGFEPDMRRLVGSPGMPTKEHRQTLMFSATYPEDIQR
Danio           GRGKIGLSKVRYLVLDEADRMLDMGFEPEMRKLVASPGMPSKEERQTLMFSATYPEDIQR
                *********:**:***************:**:**.*****:**.****************

Argy.           MAADFLKTDYLFLAVGVVGGACSDVEQTFVQVTKFSKREQLLDVLKTTGTERTMVFVETK
Danio           MAADFLKVDYIFLAVGVVGGACSDVEQTIVQVDQYSKRDQLLELLRATGNERTMVFVETK
                *******.**:*****************:*** ::***:***::*::**.**********
```

3) *Mugil cephalus*: VASA sequence not available

*Since the *Argyrosomus regius* VASA sequence is not available*,* *Danio rerio* VASA partial sequence was compared with the corresponding sequence of *Argyrosomus japonicus.* ***Danio rerio* VASA sequence provided by the manufacturer

Rabbit anti-OCT4 polyclonal antibody (Thermo Fisher Scientific Inc., PA5-27438): recombinant fragment corresponding to a region within amino acids 1 and 360 of human OCT3/4 (in grey).

OCT4 sequence not available for any of the fish species of this study

```
Homo......MAGHLASDFAFSPPPGGGGDGPGGPEPGWVDPRTWLSFQGPPGGPGIGPGVGPGSEVWGIPPCPPPYEFCGGMAYCGPQV

Homo......GVGLVPQGGLETSQPEGEAGVGVESNSDGASPEPCTVTPGAVKLEKEKLEQNPEESQDIKALQKELEQFAKLLKQKRITL

Homo......GYTQADVGLTLGVLFGKVFSQTTICRFEALQLSFKNMCKLRPLLQKWVEEADNNENLQEICKAETLVQARKRKRTSIENR

Homo......VRGNLENLFLQCPKPTLQQISHIAQQLGLEKDVVRVWFCNRRQKGKRSSSDYAQREDFEAAGSPFSGGPVSFPLAPGPHF

Homo......GTPGYGSPHFTALYSSVPFPEGEAFPPVSVTTLGSPMHSN*
```

*Human OCT4 sequence provided by the manufacturer

Mouse anti-Sox-2 (E-4) monoclonal antibody (Santa Cruz Biotechnology Inc., sc-365823): specific for an epitope mapping between amino acids 170-201 within an internal region of human Sox2 (in grey).

1) *Merluccius merluccius*: Sox2 sequence not available

2) *Argyrosomus regius:* Sox2 sequence not available

3) *Mugil cephalus* vs. *Homo sapiens**: 77% identity and 77% positivity

```
Mugil           KKDKYTLPGGLLAPAGNGMGSGVGVGVGAGLGGGVNQRMDSYAAHMNGWTNGGYGMMQDQ
Homo            KKDKYTLPGGLLAPGGNSMAS--GVGVGAGLGAGVNQRMDS-YAHMNGWSNGSYSMMQDQ
                **************.**.*.*  *********.********  ******:**.*.*****

Mugil           LSY-QHPGLNAHNPGQMQSMHRYDMSALQYNSMTSSQSYMNGSPTYSMSYSQQTTPGMTA
Homo            LGYPQHPGLNAHGAAQMQPMHRYDVSALQYNSMTSSQTYMNGSPTYSMSYSQQGTPGMAL
                *.* ********...***.*****:************:*************** ****:
```

*Human Sox2 sequence provided by the manufacturer
