# Supplementary figures and images for: Identification, Isolation, and In Vitro Culture Trials of Ovarian Germ Stem Cells from Different Teleost Fish Species
Source: Vet Sci. 2025 Dec 10;12(12):1179. doi: 10.3390/vetsci12121179 (PMC12737627; doi:10.3390/vetsci12121179)

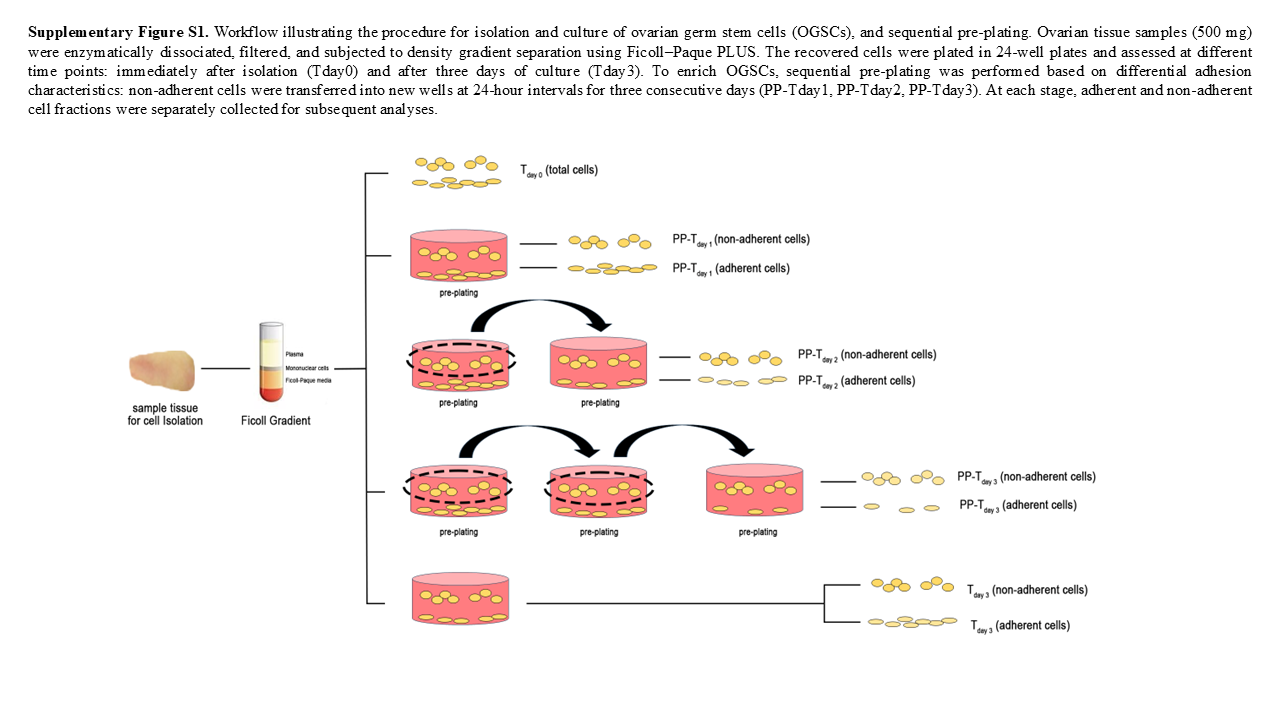

Supplement: Supplementary file 1 [file vetsci-12-01179-s001.zip › Supplementary files/Supplementary Figure S1.tif]

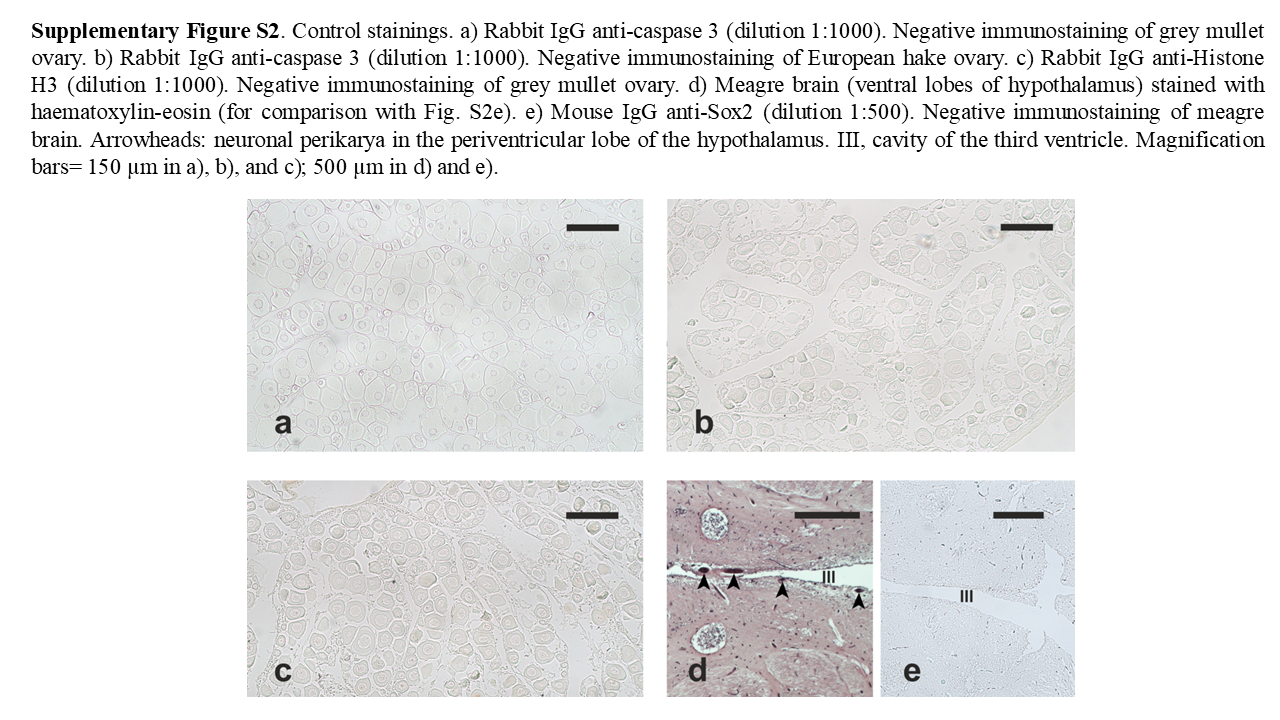

Supplement: Supplementary file 1 [file vetsci-12-01179-s001.zip › Supplementary files/Supplementary Figure S2.tif]

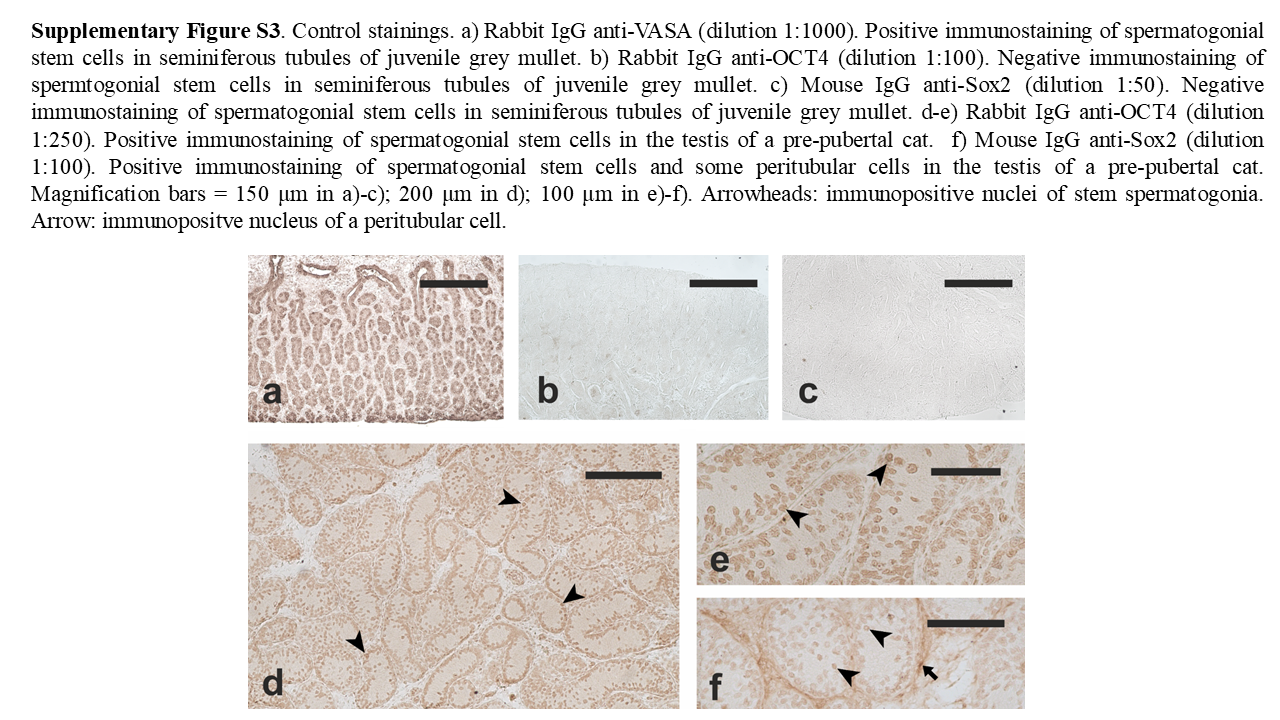

Supplement: Supplementary file 1 [file vetsci-12-01179-s001.zip › Supplementary files/Supplementary Figure S3.tif]

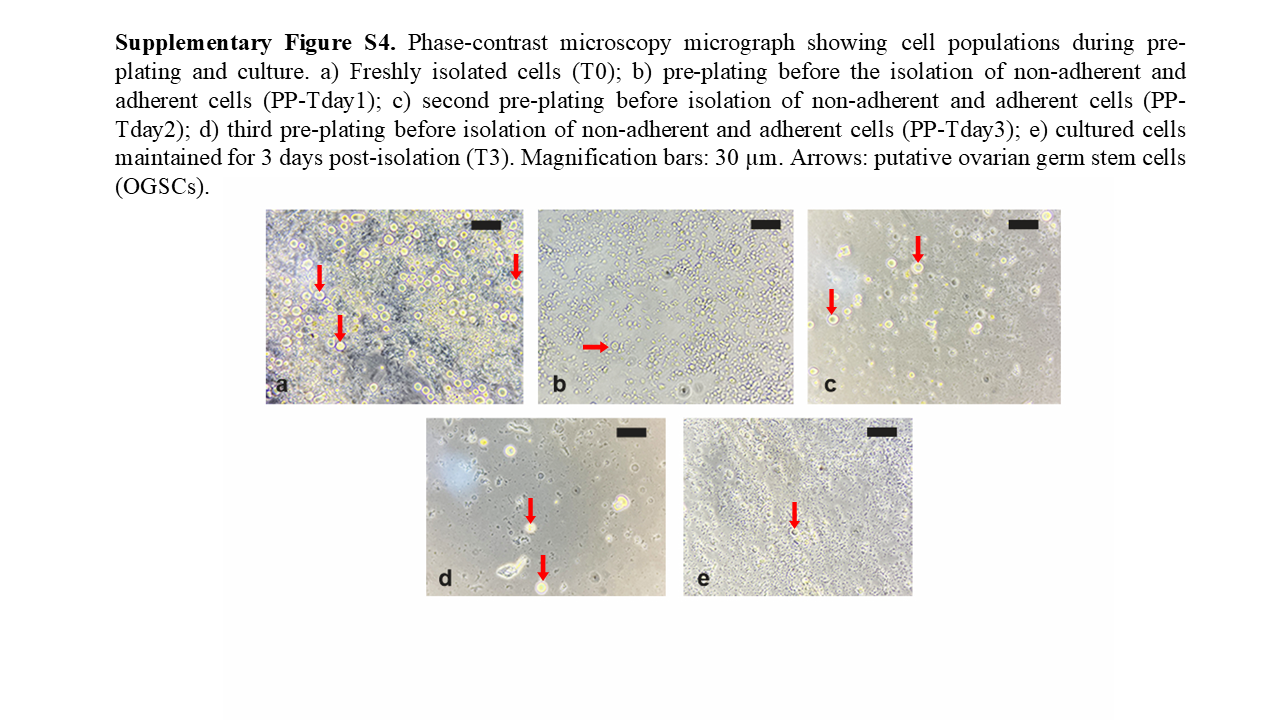

Supplement: Supplementary file 1 [file vetsci-12-01179-s001.zip › Supplementary files/Supplementary Figure S4.tif]
